# Eating disorders in adolescence: attachment issues from a developmental perspective

**DOI:** 10.3389/fpsyg.2015.01136

**Published:** 2015-08-10

**Authors:** Manuela Gander, Kathrin Sevecke, Anna Buchheim

**Affiliations:** ^1^Institute of Psychology, University of Innsbruck, Innsbruck, Austria; ^2^Department of Child and Adolescent Psychiatry, Medical University of Innsbruck, Innsbruck, Austria

**Keywords:** attachment, eating disorders, adolescence, measurement of attachment, unresolved attachment

## Abstract

In the present article we review findings from an emerging body of research on attachment issues in adolescents with eating disorders from a developmental perspective. Articles for inclusion in this review were identified from PsychINFO (1966–2013), Sciencedirect (1970–2013), Psychindex (1980–2013), and Pubmed (1980–2013). First, we will outline the crucial developmental changes in the attachment system and discuss how they might be related to the early onset of the disease. Then we will report on the major results from attachment studies using self-report and narrative instruments in that age group. Studies with a developmental approach on attachment will be analyzed in more detail. The high incidence of the unresolved attachment pattern in eating disorder samples is striking, especially for patients with anorexia nervosa. Interestingly, this predominance of the unresolved category was also found in their mothers. To date, these transgenerational aspects are still poorly understood and therefore represent an exciting research frontier. Future studies that include larger adolescent samples and provide a more detailed description including symptom severity and comorbidity would contribute to a better understanding of this complex and painful condition.

## Attachment Patterns in Eating Disorders

While eating disorders (ED) can affect individuals from different age groups, the average age of onset takes place during adolescence. During the second half of the 20th century, the prevalence rates of ED have dramatically increased and have remained relatively stable over the last 20 years (Voderholzer et al., 2012). Results from different studies looking at the prevalence of ED indicate that between 1 and 4% of adolescents meet the DSM-IV-TR criteria for anorexia nervosa (AN) or bulimia nervosa (BN) and at least 5% meet the criteria for eating disorders not otherwise specified (EDNOS; Hoek, 2006; Allen et al., 2013). Adolescents seem to be the most at-risk group to develop an eating disorder and this is due to a number of different environmental, social, psychological and biological factors. Attachment theory, originating from work Bowlby’s (1969), offers a comprehensive framework for understanding the individual and family characteristics contributing to the development of ED in this age group. Furthermore, it provides an insight into a range of different psychological functions like emotion regulation and interpersonal functioning which are relevant for ED.

Attachment has an important influence on how young people can deal with the challenging transformations during adolescence (Allen, 2008). Throughout the developmental history of an individual, secure attachment relationships provide emotional support, comfort and availability especially during stressful situations and moments of important change. When children grow older, they start to internalize daily interactions and experiences with their parents. In other words, they develop so called “internal working models of attachment” that derive from variations in how caregivers respond to their child’s attachment behavior (Bowlby, 1969). In securely attached infants, attachment events have led them to anticipate their caregivers’ availability, understanding and responsiveness. Consequently, they will experience themselves as competent and valuable. In contrast, when caregivers show a rejecting or inconsistent response to the signals of their children, they tend to experience themselves as incompetent and unlovable (George and West, 2012). According to attachment theory, a secure quality of attachment relationship is crucial in solving developmental tasks in adolescence like adjusting to physical changes, creating an own identity or defining goals for the future and thus represents an important buffer for psychological risks.

During adolescence the redefinition of the parent–child relationship in accordance with the developmental process of individuation represents one of the most challenging tasks (Allen, 2008; Dubois-Comtois et al., 2013). Teenagers achieve independence from their primary attachment figures on different emotional and behavioral levels. For example, they start to create alternative methods to deal with stress which range from relying on peers to using internal cognitive strategies. Furthermore, they make decisions independently, take actions on their own and have their personal attitudes and beliefs regarding spirituality, politics and moral values (Allen and Miga, 2010). This struggle for autonomy can be very distracting as it seems to be directly in competition with their attachment system. On the one hand they need to master new environments on their own and thus decrease their need for dependence on their parents. On the other hand they seek their parents’ comfort and solace especially under conditions of severe stress. This often paradoxical situation can lead to tremendous stress. A successful negotiation between these contradictory issues can only be achieved when both parents and their children can openly communicate their current emotional states and related thoughts (Cassidy, 2001; Allen, 2008).

Another important attachment issue of adolescence is the capacity to re-evaluate the nature of the attachment relationship with parents. When young people start to become more autonomous, they develop accurate and thoughtful responses to attachment experiences. Even if this process might be uncomfortable for parents, it is fundamental to form secure relationships with others in the future and reconsider and alter the own states of mind regarding attachment (Allen, 2008). Theorists propose that difficulties of adolescents and their parents in facilitating developmental strivings toward independence are a significant precursor for the development of an ED (Bornstein and Greenberg, 1991; Rhodes and Kroger, 1992).

The aim of this article is to review studies looking at attachment issues and their relation to adolescent ED from a developmental perspective. Additionally we will outline clinical implications of attachment-related issues in this age group which affect practice areas like psychological assessment, case formulation, therapy compliance and specialized intervention plans. A growing number of papers on attachment and ED have been published in the last decade, including some reviews (O’Shaughnessy and Dallos, 2009; Zachrisson and Skårderud, 2010; Tasca et al., 2011; Tasca and Balfour, 2014). However, research examining attachment issues in adolescents with ED have only recently begun. To date, there is no review analyzing results in adolescents with ED in particular. In the present article, we want to expand previously published reviews by reporting on findings in the field of adolescent ED with a special focus on research adopting a developmental approach. In the upcoming sections, we will offer a comprehensive overview on the latest findings from self-report and narrative-based research in adolescents with ED. For the first time, we analyze results from studies with a developmental approach on attachment in more detail. These findings will then be discussed in the context of different methods used by researchers before moving to the significance of the unresolved attachment pattern in ED. Based on these data, we identify some interesting avenues for future research and outline the clinical implications for the assessment and treatment of adolescents with ED.

## Method

The existing literature examining attachment in adolescents with ED is small but a search of current research databases (*PsychINFO*, *Sciencedirect*, *Psychindex*, and *Pubmed*) reveals that the number of papers looking at this age group specifically has increased in the last years. Articles for inclusion in this review were identified from PsychINFO (1966–2013), Sciencedirect (1970–2013), Psychindex (1980–2013) and Pubmed (1980–2013). The search terms “*adolescence*,” “*eating disorders*,” “*anorexia nervosa*,” and “*bulimia nervosa*” were used as major descriptors. We only included articles which (1) stated the use of the search terms in the title, key words or abstracts, (2) are published in the English or German language in a peer-reviewed journal or book chapter and either (3) used a clinical sample including adolescents (≤ 18 years) or young adults or (4) a non-clinical adolescent sample with significant eating problems that are defined as sub-clinical degrees of disturbed eating behaviors and attitudes as well as body and weight dissatisfaction. As we need to keep the scope of this review contained and manageable, we only included studies using self-report questionnaires of attachment style (e.g., The Attachment Style Questionnaire; Experiences in Close Relationships Questionnaire) and narrative attachment measurements (AAI, AAP). We excluded studies that (1) only use self-report questionnaires assessing parental styles (e.g., The Parental Bonding Instrument) as they measure a concept that is different from attachment styles and patterns (for a recently published review on parental bonding in patients with ED, see Tetley et al., 2014) (2) focus on the period of childhood and preadolescence (< 12 years) (3) only include adult or older patients (> 24 years). Furthermore, we focus on AN, BN and EDNOS as the primary conditions in adolescents and therefore, we excluded studies solely concentrating on obesity, binge eating disorder (BED) or feeding disorders in childhood. Using the search terms mentioned above we found 81 published studies and 5 reviews on ED and attachment. From these 81 studies, 12 studies employ narrative techniques and 69 use self-report questionnaires to measure attachment. Furthermore, we identified 21 studies including an adolescent sample that will be reviewed in more detail. First, we will present age-specific findings from self-report adolescent studies and incorporate these results into the large body of research in adults. Then we will focus on the developmental approach and report on the major results concerning the unresolved attachment representation. Finally, we will outline implications for therapeutic intervention in adolescents and describe some interesting avenues for future attachment research in that age group.

## Adolescent Attachment and ED Using Self-report Questionnaires

In recent years, an increasing number of researchers examining attachment in adolescents with ED have come to rely on self-report questionnaires of attachment. Self-report questionnaires either assess categories of attachment style or they measure the degree to which dimensions of attachment styles are present (Ravitz et al., 2010). The outcome of self-report items is a product of thoughts about attachment that have entered the consciousness of a person and therefore reflect how the individual wishes to represent him- or herself toward others. Several self-report measurements like for example the Adult Attachment Questionnaire and the Experiences in Close Relationships converge on two dimensions of insecurity. Attachment anxiety refers to individuals with a negative sense of the self. They tend to expect separation, abandonment or insufficient love and they are preoccupied with the availability and responsiveness of others. Furthermore, they tend to maximize negative experiences, they are hypervigilant to potential threat and they demonstrate a hyperactivation of attachment behavior. In contrast, attachment avoidance refers to individuals with a negative sense of others. They are characterized by self-reliance, an avoidance of intimate relationships and they devaluate the importance of close relationships. In addition, they minimize feelings of distress and deactivate attachment behavior. To date, attachment studies using self-report questionnaires in adolescents have addressed fundamental questions about possible links between attachment styles, ED subtypes, symptom severity and treatment outcome.

The overall patterns of results across different studies provide strong evidence that there is a relationship between attachment classifications and ED. Collectively, findings from studies using self-report attachment questionnaires assessing secure, avoidant, anxious and for some measures fearful attachment styles (i.e., high insecurity on both attachment avoidance and anxiety) suggest that adolescents and young adults with ED have an insecure attachment style (Orzolek-Kronner, 2002; Steins et al., 2002; Bäck, 2011). Some authors found a higher prevalence of the avoidant attachment style (Ramacciotti et al., 2001; Latzer et al., 2002) and others found more anxious attachment styles in adolescent patients with ED (Salzman, 1996; Tereno et al., 2008). Some authors speculated that an anxious attachment style characterized by a tendency for affective dysregulation might be associated with binge eating and purging behavior whereas avoidant attachment style characterized by a tendency to down regulate emotions might be linked to dietary restriction. However, the study results are not consistent concerning these associations suggesting that the diagnostic subgroup is not necessarily related to attachment insecurity dimensions (Troisi et al., 2005; Strauss et al., 2006; Tereno et al., 2008; Illing et al., 2010; Dakanalis et al., 2014).

Thus, recent work in this field started to rather investigate links between adolescent attachment styles and symptom severity. This approach holds a good deal of promise for advancing our understanding of the psychopathological profile of patients with ED. Studies in young adults with AN and BN have already demonstrated that higher attachment anxiety was significantly related to greater ED symptom severity and poorer treatment outcome in patients with AN and BN (Cash and Annis, 2004; Illing et al., 2010). A recently published study by Keating et al. (2015) has expanded this research by examining the influence of pre-treatment attachment insecurity on post-treatment depressive symptoms in adolescent and adult patients with ED. In their study, patients with a pre-treatment anxious attachment style demonstrated less reduction in depression than those with an avoidant attachment style. These results indicate that the increased need for approval and the hyperactivation of emotions in anxiously attached patients may impair their ability to develop useful coping strategies leading to a comparison with unrealistic standards. This is consistent with results from a growing number of studies demonstrating that hyperactivating strategies are mediators for attachment anxiety and depressive symptomatology in adolescents and adults (Tasca et al., 2009; Malik et al., 2014). Even though these findings suggest that attachment anxiety influences symptom severity, only very little is known about the role of an insecure attachment style on the ED symptom change during and after treatment. Thus an interesting avenue for future research is to examine attachment-related predictors for short- and long-term outcomes in adolescents with AN. Furthermore, the aforementioned studies assessed attachment style only at pre-treatment and as studies from adults demonstrate that attachment can change after treatment it would be interesting to measure it at post-treatment as well (Buchheim et al., 2008, 2012a; Smith and George, 2012; George and Buchheim, 2014; Salcuni et al., 2014).

In addition to these possible links between attachment styles, symptom severity and treatment outcome, researchers have become increasingly interested in how far attachment style represents a potential risk factor for developing an ED in adolescence. According to attachment theory, attachment styles have lasting implications for social information processing, emotion regulation and self-evaluative processes making individuals vulnerable to ED. Milan and Acker (2014) found that attachment insecurity is related to increased responsivity to individual (e.g., weight gain) and interpersonal (e.g., maternal negative affect) ED risk factors during adolescence. However, only few studies to date have addressed specific underlying cognitive and emotional processes explaining why attachment insecurity places adolescents at a greater risk for developing an ED. Demidenko et al. (2010) found that insecure attachment in adolescents is related to a poorer self-concept and lower identity differentiation—two dimensions commonly known as risk factors for developing an ED. Mindfulness seems to be another mechanism underlying this relationship (Pepping et al., 2015). That is, individuals with an insecure attachment style are less capable to be aware of and accept the present moment without judging it leading to an impaired recognition of hunger and satiety as well less acceptance of the body and the self. Additionally, maladaptive perfectionism and problematic affect regulation seem to mediate the relationship between attachment insecurity and ED in adults (Tasca et al., 2009; Dakanalis et al., 2014). To draw further conclusions on causal relationships, future studies should examine these associations longitudinally and find out in how far findings vary according to the stage of recovery. As all of the previously mentioned studies only include female participants, it remains unknown whether the same patterns of results would be observed in males.

In the last decades, a growing body of researchers has examined the role of the family for adolescents with ED. Some studies investigated in how far the relationship between parents and their children are related to the development of an ED in adolescents (Troisi et al., 2005; Bäck, 2011). In one of the first studies examining attachment phenomena in adolescents with ED, Kenny and Hart (1992) found that parental fostering of autonomy and affectively positive and emotionally supporting parental relationships lead to less weight preoccupation, feelings of ineffectiveness and bulimic behaviors in young people. These results are consistent with subsequent literature findings concerning characteristics of parental relationships in ED patients (Latzer et al., 2002; Bäck, 2011). Concerning the issue of parental relationships, Orzolek-Kronner (2002) hypothesized that ED behaviors like starvation, binge eating and purging might lead to close physical encounters between mothers and their daughters and thus increase physical and psychological proximity. In her study, adolescents with ED reported more proximity seeking behaviors than the clinical and non-clinical control groups. One unexpected finding emerging from this study was that adolescents with ED viewed their mothers as greater facilitators of independence compared to the control groups. This finding seem to be contradictory to earlier results demonstrating that undermining an adolescent’s autonomy striving might lead to more body dissatisfaction and greater risk for the development of ED symptoms (Kenny and Hart, 1992; Latzer et al., 2002). Furthermore, there seems to be evidence of maternal and paternal overprotection and a higher amount of fragile, dependent mothers who strive toward a symbiosis with their ED daughters (Tereno et al., 2008; Amianto et al., 2013). Thus the description of their mothers as supporting their autonomy might reflect the adolescents’ attempts to preserve the idealization of their primary caregiver. The idealization of parents is considered as a common feature of an avoidant attachment style and might explain why some authors have found a higher prevalence of this style in adolescent patients with ED (Latzer et al., 2002).

In sum, the results found in these studies highlight the importance of attachment-related aspects for the etiology, psychopathological profile, treatment outcome and parental relationships in adolescents with ED (for details on the sample and measurements see Table 1).

**TABLE 1:** **Studies using self-report questionnaires in a sampleincluding adolescents with ED**.

**References**	**Sample**	***N***	**Gender**	**Age**	**Aim of the study**	**Measurement**
Pepping et al. (2015)	Adolescent and adult undergraduates	144	Female	17–40	To investigate whether mindfulness is a mechanism underlying the relationship between greater eating pathology and attachment insecurity	ECR
Milan and Acker (2014)	Adolescents from a community sample	659	Female	14–15	To examine in how far early attachment style moderates ED risk among adolescent girls	BSQ
Keating et al. (2015)	Out-patients with AN (*n* = 54) and BN (*n* = 87)	141	Female	14–40	To study in how far pre-treatment attachment insecurity predicts changes of post-treatment ED and depressive symptoms	ASQ
Amianto et al. (2013)	Daughters with AN (*n* = 81), BN (*n* = 31) and EDNOS (*n* = 61) and their parents	173	Female	16–45	To explore attachment style and other character traits in parents of daughters with ED	ASQ
Bäck (2011)	High school students from a community sample	80	Mixed	17–18	To examine the effects of parent–child relationship quality and upbringing in food situations on eating problems	Modified version of the RQ
Demidenko et al. (2010)	Women with AN (*n* = 64), BN (*n* = 133), EDNOS (*n* = 96), BED (*n* = 37)	330	Female	17–37	To investigate in how far self-concept mediates the relationship between attachment insecurity and identity differentiation	ECR
Tasca et al. (2009)	Out-patients with AN (*n* = 74), BN (*n* = 138), EDNOS (*n* = 98)	310	Female	17 and older	To investigate the role of affect regulation strategies in mediating the relationship between attachment dimensions and ED and depressive symptoms	ECR
Tereno et al., 2008)	In-patients with AN (*n* = 30) and BN (*n* = 27) and healthy controls (*n* = 35)	92	Female	15–30	To investigate differences in attachment style and perception of memories of parental rearing between AN, BN and healthy controls; comparison of parent’s and daughter’s attachment styles; influence on the therapeutic bond	AAS-R
Troisi et al. (2005)	Out-patients with AN (*n* = 19), BN (*n* = 31) and EDNOS (*n* = 19) and healthy controls (*n* = 64)	133	Female	17–36	To assess prevalence rate of attachment styles in the ED group; relationship between symptoms of separation anxiety in childhood, attachment style and demographic variables (age, educational level, age of onset, illness duration)	ASQ
Hochdorf et al. (2005)	In-patients with AN (*n* = 34) and BN (*n* = 34), healthy controls (*n* = 37)	105	Female	15–29	To find out how attachment styles are related to attraction to death among ED patients	AAS
Latzer et al. (2002)	In-patients with AN (*n* = 25) and BN (*n* = 33), 23 healthy controls	81	Female	15–30	To examine the extent to which family environment factors (cohesiveness, expressiveness, encouragement of personal growth) and attachment styles are associated with ED	AAS
Orzolek-Kronner (2002)	Adolescents with ED (*n* = 44), with varying DSM-IV diagnoses (*n* = 28) and healthy controls (*n* = 36)	108	Mixed	14–18	To establish a link between parental attachment, proximity seeking and the development of an ED	IPPA (inventory of parent and peer attachment), PAQ^k^
Kenny and Hart (1992)	In-patients with AN (*n* = 18) and BN (*n* = 50), 162 healthy controls	230	Female	15–19	To analyze the relationship between parental attachment and ED symptoms	PAQ (parental attachment questionnaire)

ECR, experiences in close relationships questionnaire; BSQ, behavioral systems questionnaire; AN, anorexia nervosa; BN, bulimia nervosa; ASQ, attachment style questionnaire; EDNOS, eating disorders not otherwise specified; RQ, relationships questionnaire; BED, binge eating disorder; AAS-R, adult attachment scale- revised; AAS, adult attachment scale; IPPA, inventory of parent and peer attachment; PAQ, parental attachment questionnaire.

While these investigations look at thoughts about attachment that have entered the consciousness of a person and therefore reflect how adolescents perceive themselves in relationships, they are not concerned with unconscious defensive processes and strategies which are essential for the developmental approach (Ravitz et al., 2010; George and West, 2012). Using narrative techniques to assess developmental attachment patterns allows us to look beyond conscious thoughts of relationships and evaluate mental representations of attachment by analyzing patterns of responses when people talk about attachment situations. Employing these techniques might provide a deeper understanding of unconscious aspects of attachment-related defenses and behaviors and allow assessing attachment disorganization and trauma (George and West, 2012; George and Buchheim, 2014).

## Adolescent Attachment and Eating Disorders Using Narrative Techniques

The majority of the studies on attachment in adolescents and young adults with ED are using self-report measures and only very few make use of narrative techniques. The adult attachment interview (AAI; George et al., 1996) and the adult attachment projective picture system (AAP; George and West, 2001, 2012) are two narrative interviews, which emphasize mental representations. These techniques allow analyzing unconscious defensive processes—a dimension which is lost in self-report measures. In the AAI individuals are asked to describe childhood and current experiences with their caregivers and recall specific attachment-related events. Secure individuals (F) are able to reflect and integrate positive and negative experiences with their caregivers and their evaluation of attachment experiences is coherent. In contrast to them, insecure-dismissing individuals (Ds) tend to idealize or devaluate their attachment experiences by deactivation of attachment distress. The insecure-preoccupied (E) group is enmeshed with their caregivers and they show anger and low autonomy in their narrative evaluation. Finally, the unresolved category (U) refers to individuals who show a breakdown of defensive and coping strategies when talking about traumatic experiences like loss and abuse. Their evaluation is incoherent and often includes fearful affect (Buchheim and George, 2011, 2012). The AAP is a narrative-based attachment measure that provides attachment classification based on the analysis of “story” responses to a set of theoretically-derived attachment-related drawings of scenes depicting solitude, illness, separation, death, and potential maltreatment. The AAP narratives of secure individuals (F) demonstrate the ability and willingness to think about attachment distress. In their stories characters reach out to attachment figures for comfort, they show a lot of constructive actions and they often describe mutual enjoyment in their relationships to others. The insecure-dismissing (Ds) group is characterized by a predominance of deactivating defensive processes that emphasize distance in relationships. Their narratives often focus on achievement and exploration. Attachment relationships usually provide functional care or they are described as authoritarian. The AAP stories of insecure-preoccupied (E) individuals include a lot of material that confuses and obscure attachment relationships. They typically concentrate on emotions related to problems, their responses have several undecided themes or story endings and they often focus on the past rather than on the present. This confusion in the story line is also reflected in the blurring of the hypothetical story with personal experiences. The unresolved attachment (U) refers to a group of individuals who are not able to contain and reorganize stories including features that evidence danger, helplessness, failed protection or isolation. Unresolved individuals become momentarily flooded by their attachment fears that cannot be reorganized in the narratives (George and West, 2012).

To our knowledge, there are only eight studies using the AAI or the AAP in a sample including adolescents and young adults with ED (Cole-Detke and Kobak, 1996; Salzman, 1996; Ramacciotti et al., 2001; Ward et al., 2001; Dallos and Denford, 2008; Dias et al., 2011; Lis et al., 2011; Sevecke, 2013) and interestingly, there are only three studies looking explicitly at adolescents (Dallos and Denford, 2008; Lis et al., 2011; Sevecke, 2013). In the following paragraphs we are going to outline the main results of these studies by focusing on attachment strategies and underlying neurophysiological correlates in adolescents with ED before moving to the significance of the unresolved attachment status for this age group (for details on the sample and measurements see Table 2). In the final part of this section we discuss implications of these findings for future research.

**TABLE 2:** **Studies using narrative techniques in a sampleincluding adolescents with ED**.

**References**	**Sample**	***N***	**Gender**	**Age**	**Aim of the study**	**Measurement**
Sevecke (2013)	Adolescents with AN	34	Female	13–17	To examine attachment representations and personality disorders, self-harming behaviors, identity problems and emotional dysregulation	AAP
Dias et al. (2011)	Women with AN (*n* = 28), BN (*n* = 15), EDNOS (*n* = 4)	47	Female	15–36	To investigate attachment representations and autonomic response during attachment interview	AAI
Lis et al. (2011)	Single case study of an anorexic young woman	1	Female	17	To integrate an attachment tool in a multimethod assessment and intervention plan	AAP
Dallos and Denford (2008)	Families: daughters with AN (*n* = 4)	4	Female	16–19	To explore current relationships and attachment themes in families	AAI (modified version)
Ward et al. (2001)	In-patients with AN (*n* = 20) and their mothers (*n* = 10)	20	Female	15–46	To assess attachment representations in mothers and daughters	AAI
Ramacciotti et al. (2001)	Outpatients with AN and EDNOS: females (*n* = 7) and males (*n* = 6)	13	Mixed	17–36	To determine states of mind regarding attachment in ED patients	AAI
Cole-Detke and Kobak (1996)	College women (*n* = 44) with ED symptoms	44	Female	18	To examine attachment strategies in adolescents with higher and lower levels of depressive and ED symptoms	AAI
Salzman (1996)	28 female undergraduates: histories of AN (*n* = 7)	7	Female	18–22	To study links among attachment representations, affective instability and ED	AAI

AN, anorexia nervosa; AAP, adult attachment projective picture system; BN, bulimia nervosa; EDNOS, eating disorders not otherwise specified; AAI, adult attachment interview.

In narrative-based attachment research, a number of studies have addressed the issue of attachment patterns in adolescents with ED using the AAI. Salzman (1996) investigated links among attachment patterns, affective instability and ED in young college women. Her most striking observation was that the insecure-dismissing attachment group showed the highest prevalence of ED. The dismissing daughters described the nature of the attachment to their mothers as “hot and cold,” “addictive love,” and a “push and pull relationship.” During the AAI, they focused on their mothers’ emotional inconsistency and they held their mothers responsible for their own distress. Furthermore, they mentioned unpredictable and hurtful attacks of their mothers on their self-image and social acceptability. At the same time they talked about moments of true understanding, i.e., when mothers are caring and offering the promise of a special connection. This paradox of longing for their mother on the one hand, and rejecting her on the other hand might be the trap that leads to affective dysregulation and an eating disorder. The starving might serve as a kind of nurturance that they cannot get from their mothers. It must be noted that although these results are based on a very small sample (*n* = 7), they are consistent with the Ward et al. (2001) and Ringer and Crittenden (2007) findings of a push- and pull strategy for both denying any need for help and also seeking care. This interesting association between attachment patterns in adolescents and ED led to further investigations on attachment strategies in ED patients that define how individuals process distress-related memories in the AAI.

For example, Cole-Detke and Kobak (1996) examined attachment strategies in a sample of 44 college freshmen with clinically significant ED symptoms.. While a secure strategy means that a person can adaptively tolerate distressing childhood memories, defensive strategies develop when attachment figures are unavailable, insensitive or unresponsive. Researchers distinguish two subtypes of defensive strategies. The deactivating strategy develops when an individual perceives an attachment figure as rejecting or ignoring and therefore has to divert attention away from attachment distress. The hyperactivating strategy develops when attachment figures are perceived as inconsistently responsive and therefore individuals excessively focus on attachment-related information during the AAI which then results in passive or angry preoccupation (Dias et al., 2011). When depression was statistically controlled, the authors found that young women with deactivating strategies were prone in reporting elevated levels of ED symptoms. It is assumed that these women have to shift their attention away from attachment toward more attainable goals like one’s appearance in order to win the approval of their emotionally unavailable and highly critical fathers. There are a couple of noteworthy limitations to this study. First of all, the study included a subclinical sample which limits the generalizability of the results. Second, the predominance of deactivating strategies among women with ED is mostly explained by the poor relationships to fathers but not to the mothers. Third, we have to keep in mind that a significant amount of women with ED symptoms also met depressive criteria (*n* = 19). As there is a high comorbidity rate of depression in patients with ED, which would be associated with hyperactivating strategies during the AAI, future studies should address if there are differences between ED with and without psychiatric comorbidity in adolescence (Salbach-Andrae et al., 2008; Hughes et al., 2013).

Underlying autonomic parameters of these attachment strategies in adolescents were investigated by Dias et al. (2011). In their study they examined the physiological response of 47 women including adolescents with ED during the AAI. Interestingly they found that hyperactivating strategies were rather associated with the purging/binge eating group (BN, AN-binge eating/purging, BED and EDNOS with “chaotic” eating behavior) than with the restrictive group (AN-restrictive, EDNOS with high control/restrictive features). Concerning attachment patterns, 70% of the participants were classified as insecurely attached with a higher proportion of preoccupied individuals. On a physiological level these individuals displayed a higher electrodermal reactivity and cardiac reactivity when compared to the securely attached group. These results go in line with a number of other studies investigating the neurophysiological correlates of attachment during the AAI (Beijersbergen et al., 2008; Holland and Roisman, 2010; Gander and Buchheim, 2015). The underlying autonomic parameters might suggest that patients with ED and an insecure attachment pattern feel more challenged when talking about early experiences. Furthermore, their emotion regulation strategies might be less productive when confronted with their own attachment experiences with caregivers. Interestingly, the heart rate variability was significantly correlated with attachment insecurity when they were asked to name five adjectives about the relationship with their fathers. Although these results extend previous work on autonomic correlates of adult attachment by including a clinical sample of patients with ED, the results must be interpreted with caution. The authors did not control for psychiatric medication which potentially could influence the physiological parameters and they did not use a control group to compare the results. Furthermore, both of the above mentioned studies did not include the unresolved category into their research. When looking at disorganized infants in the Strange Situation, we observe that they suffer from tremendous stress as indicated by high cortisol levels, increased heart rate and skin conductance (Bernard and Dozier, 2010). Therefore an interesting avenue for future psychobiological attachment research is to identify and connect the moment of breakdown in unresolved individuals with recordings of physiological reactivity.

Currently, there are only a handful of published studies including the unresolved attachment category in a clinical sample of ED. In the following paragraphs we are going to discuss the main results of these studies and present one of the only studies focusing explicitly on adolescents.

### The Role of the Unresolved Attachment Status (U) for ED

Narrative-based attachment studies indicate a high prevalence rate of the unresolved attachment category and provide an interesting insight into the nature of these traumatizing events. Furthermore, first studies demonstrate how attachment issues can be integrated into a multimethod assessment and be used for interventions in ED. Before launching into characteristics of the unresolved attachment classifications and its implications for the treatment in adolescents with ED, it is necessary to explore the role of trauma for this ED in general. It is well known that sexual and physical traumatization represent non-specific risk factors for the later development of an ED (Jaite et al., 2012; Lejonclou et al., 2014). Practitioners who treat patients with ED often encounter histories of various traumatic and abusive experiences. With regard to the whole spectrum of traumatic experiences, patients with ED frequently report interpersonal trauma and adverse childhood circumstances like emotional abuse, mental health problems in parents or parental divorce during their upbringing (Lejonclou et al., 2014). The results of different studies suggest that sexual trauma is associated more often with BN, AN-binge-eating/purging type and BED than with AN-restrictive type (Brewerton, 2007; Jaite et al., 2012). However, only very little is known about the relationship between early traumatizing events and ED.

When it comes to attachment research, there are only very few studies looking at unresolved attachment representation in ED. In summary, studies including the unresolved attachment status indicate a predominance of this category in ED (Ward et al., 2001; Ringer and Crittenden, 2007; Sevecke, 2013; Delvecchio et al., 2014; Von Wietersheim et al., 2014). One of the first studies examining the prevalence of an unresolved attachment status (U) was done by Fonagy et al. (1996). They investigated the relationship between attachment patterns and psychiatric patients and found a high prevalence of U with respect to loss and abuse in the ED group. Although the sample size (*n* = 14) is very small, it is an interesting result that 13 of the patients were classified as U when using the four-way attachment distribution of the AAI. Additionally, they found a high idealization of parents and low reflective functioning (i.e., the capacity to understand and reflect on mental states of the self and others) in these patients. When talking about attachment experiences, they never reflected on mixed emotions, conflict or uncertainty about feelings of others. Furthermore, their responses appeared somewhat clichéd, banal or superficial. This high prevalence rate of the U category could also be found in other studies using the AAI (Ward et al., 2001; Zachrisson and Kulbotten, 2006; Ringer and Crittenden, 2007; Barone and Guiducci, 2009) and the AAP in anorexic and bulimic adolescent and adult samples (Delvecchio et al., 2014; Von Wietersheim et al., 2014). In the study of Delvecchio et al. (2014) half of the adult anorexic patients were classified as U and 37% were classified as Ds. Interestingly, Sevecke (2013) assessed attachment representations with the AAP in inpatient adolescent girls with AN (*n* = 34) and found that even 65% had an unresolved attachment status. These findings might suggest that adolescents with ED have an even higher prevalence of the U than adults.

A growing body of research has made notable strides in understanding specific characteristics of the unresolved attachment status by analyzing the narratives of ED patients. Delvecchio et al. (2014) found that the majority of anorexic women with an insecure or unresolved attachment classification reported a lot of traumatic material including eerie, evil or surreal elements in their response to the AAP picture stimuli. Ringer and Crittenden (2007) analyzed the content of AAI narratives in a larger sample of women with ED (*n* = 67) and found a lack of resolution of trauma and loss related to the mothers and also hidden family conflict between the parents. One third of the daughters with ED were classified as unresolved concerning the following aspects: (1) fighting between the parents, (2) vicarious experience of a parent’s trauma or (3) an imagined relation between an event and ED. This is consistent with the results of the Dallos and Denford (2008) study. When examining the main areas of unresolved and traumatic processes in four families with anorexic daughters, they observed that one of their female anorexic adolescents showed a terrible fear that her parents might do something awful to each other. Yet interestingly, also the mother of this girl reported unresolved traumatic memories of her own depressed mother and her angry, unpredictable and abandoning father. Similarly, most of these unresolved traumas in the Ringer and Crittenden (2007) study were not based on a direct threat to the woman herself. Unfortunately, the parents’ AAI were not available for this study. However, in the discussion the authors comment on interviews of parents outside this sample that they have read. Surprisingly, the parents appear responsible and really caring for their daughters. However, during the AAI their mothers reported losses and severe dangers in their past (e.g., war, repeated sexual abuse, loss of a parent). The most outstanding feature of their discourse was their strong desire that their daughters are not affected by this. In other words, they wanted to protect their daughters from these unspeakable threats and disappointments of their own lives.

Ward et al. (2001) examined the attachment representations in mothers and their daughters with ED. Although this study had a small sample size (daughters *n* = 20, mothers *n* = 10), the high rate of the U category (67% of the mothers and 50% of the daughters) with respect to loss, particularly among the mothers, is quite striking. Now the question is in how far this affects the mother’s behavior. It can be hypothesized that although they seek a special closeness to their daughters and protect them from their own traumatic histories, they retreat or become unavailable in crucial moments when their daughters need them the most. Hardly anything is known about the nature of these traumatizing experiences in mothers. One possible traumatizing event in mothers was found by Shoebridge and Gowers (2000). They observed a high frequency of obstetric losses (e.g., multiple miscarriages, stillbirth, early neonatal death) prior to the birth of their daughters who later on developed AN. Furthermore, there are no published studies looking at attachment using the AAI in fathers of these daughters with ED. Therefore an interesting avenue for future research would be to investigate a larger number of mother-daughter pairs/father-daughter pairs looking for transgenerational similarities. In addition, Barone and Guiducci (2009) found the unresolved attachment status only in patients (*n* = 30) with BN and BED but not in AN. However, this unequal distribution also requires a replication using a larger sample to draw further conclusions.

Another interesting aspect is the integration of an attachment tool in a multimethod assessment in patients suffering from an ED. Lis et al. (2011) discussed the clinical implications using an attachment tool for a better etiological understanding and treatment plan. They presented a single case study of a 17-year old anorexic girl, who was classified as unresolved on the AAP. The in-depth analysis of the AAP indicates that she could not integrate and reorganize the pain and isolation she felt in relation to her illness and also the inability of her parents to provide comfort. Based on this preliminary finding, it would be an interesting avenue to analyze disorganized specific characteristics for ED patients and see in how far they differentiate from other psychiatric disorders. First studies looking at these aspects were already done for other psychiatric disorders like borderline personality disorder (PD), anxiety disorder, depression, PTSD and schizophrenia (Buchheim et al., 2008; Buchheim and George, 2011; Juen et al., 2013). Such investigations would make important contributions to our understanding the complex etiology of ED in adolescence.

## Discussion

The aim of this article was to review the literature which examines the associations between attachment patterns and ED with a focus on adolescence. This approach is still a relatively untapped area of research. Most of the studies focusing on adolescents are using self-report measures, which directly assess an individual’s conscious appraisals of feelings and behaviors in close relationships but they cannot evaluate defensive processes when participants talk about their attachment experiences. In contrast, studies employing the AAI are underrepresented in the literature and they often only include small sample sizes as the interview procedure and the coding of this narrative instrument are very time-consuming. Another issue concerning the AAI is that it is valid for assessing loss through death and physical abuse which are important aspects of the unresolved attachment status. However, according to the AAI instruction guidelines interviewers rightfully should not ask for traumatic material that interviewees do not want to talk about. That is, if an interviewee fails to discuss traumatic experiences these are edited out of the AAI transcript (George and West, 2011). The AAP is another narrative instrument that circumvents some of these potential practical drawbacks of the AAI as picture scenes serve as stimuli for the individual narratives and thus frightening memories might come up in the stories without being articulated as the interviewee’s own experiences (George and Buchheim, 2014). In addition the AAP requires less time for administration and coding but at the same time shows an impressive agreement to the AAI. It also demonstrated acceptable validity for assessing attachment representation in adolescents and thus is amenable for use in a wide range of clinical settings including younger patients (Buchheim et al., 2014).

Even though attachment is defined differently by the two approaches, the overall pattern of results from adolescent and adult samples suggest that most of ED patients are insecurely attached. One of the most striking results that emerge from narrative-based studies is the high prevalence of the unresolved attachment status in patients with ED and their mothers (Ward et al., 2001; Ringer and Crittenden, 2007; Barone and Guiducci, 2009). This is particularly important for adolescents, as there seems to be considerable evidence for a higher prevalence of the unresolved attachment status in adolescent samples (Sevecke, 2013) compared to adults (Ringer and Crittenden, 2007; Lunn et al., 2012; Delvecchio et al., 2014). Research has already demonstrated that a mother’s unresolved trauma may lead to an impaired ability to sensitively respond to her child and thus may contribute to the transgenerational transmission of trauma (Iyengar et al., 2014). However, the nature of these traumatizing experiences related to attachment is still an untapped area of research. Hence, a key priority for future research is to examine the nature of the trauma not only in adolescents with ED but also in their mothers. These investigations might broaden our understanding of the role of unresolved attachment status for an early onset of ED. Given that traumatized mothers also tend to superimpose their own needs on those of their daughters, one might expect that daughters remain dependent on their mothers to supply a sense of self as they have never learned to differentiate their own needs. However, studies unexpectedly found that adolescents with ED tend to idealize their parents and view their mothers as great facilitators of independence (Orzolek-Kronner, 2002). In contrast, attachment studies in adults found more subjectively reported problematic mother-daughter relationships. They are characterized by a loving but at the same time neglecting, rejecting and role-reversing stance leading to an exaggerated display of angry feelings, an angry demand of the caregiver’s compliance and confusion regarding the parental behavior in their adult daughters (Ringer and Crittenden, 2007; Barone and Guiducci, 2009). Replicating these findings using a longitudinal design would clearly be a promising area for future studies. Additionally, it appears that the role of fathers has largely been neglected in most of the studies using narrative techniques. A small number of studies have examined the attachment to fathers by using self-report measures (e.g., Parental Attachment Questionnaire, Parental Bonding Instrument, Inventory of Parent and Peer Attachment) in an adolescent sample with ED. The main findings show that patients feel more alienated from their fathers and they describe them as less caring and more controlling (Orzolek-Kronner, 2002; Fujimori et al., 2011; Pace et al., 2012; Horesh et al., 2015). Therefore, it requires future studies including the fathers of patients to draw further conclusions on the dyadic nature of attachment in ED.

The question why attachment insecurity or disorganization places adolescents at a greater risk for developing an ED has received increasing attention in recent years. A growing number of studies found that insecure attachment style is related to a poorer self-concept, lower identity differentiation, lower acceptance of the own body and self and an impaired recognition of hunger and satiety. Regarding these risk factors in adolescence, studies from adults with ED demonstrate that additionally maladaptive perfectionism and personality characteristics like neuroticism and extraversion that manifest during early adulthood significantly mediate the relationship between insecure attachment style and ED in adults (Eggert et al., 2007; Dakanalis et al., 2014). Future research on adolescent ED would profit by relating age-specific risk factors to different attachment patterns. An interesting question for subsequent studies is whether adolescents with an unresolved attachment status show a different amount and quality of risk factors that places them at a greater risk for an early onset than those who are insecurely attached. Furthermore, the higher prevalence of insecure attachment in adults raises fascinating questions about the role of romantic relationships for this age group. In how far attachment-related difficulties in intimate relationships represent potential risk factors or facilitate the maintenance of ED in adulthood has largely been unexplored in the research literature and thus represents an exciting research frontier for future studies (Evans and Wertheim, 2005).

Another interesting question is in how far attachment classification can be linked to diagnostic subgroups of ED. One of the major problems is that past studies on attachment have tended to focus exclusively on AN and BN but studies on BED and obesity in adolescence are still rare. Moreover, the research to date demonstrates very controversial results due to the different methods employed in the studies. As the self-report questionnaires and the narrative instruments define attachment classifications in a different way, future studies should be careful when comparing their results as this might lead to confusion of terminology (Ward et al., 2001; Ravitz et al., 2010; George and West, 2012). As suggested by O’Shaughnessy and Dallos (2009) it would be more helpful to examine if there is an association between attachment patterns and the symptom severity in ED. Yet, if future research is to identify these possible correlations we must also consider the high prevalence rate of comorbid disorders in ED like depression, anxiety and PD. In a very recent review on PD in adolescents with ED, Magallón-Neri et al. (2014) found that between 64 and 76% of adolescents with BN had a comorbid borderline PD or histrionic PD. In patients with AN the most common PD was the anankastic PD. Studies have demonstrated that the unresolved attachment classification predominates in borderline PD patients and thus the presence of a comorbid borderline PD might also have a significant influence on the attachment status of a patient with an ED (Buchheim et al., 2008). Similar results were found in a preliminary study by Sevecke (2013) who investigated associations between attachment representations and PD in 34 adolescent patients with AN. Interestingly 65% of the girls were classified as unresolved on the AAP and even 82% fulfilled the criteria for a PD. The unresolved group showed a significantly higher PD comorbidity rate when compared to the other attachment groups. Furthermore, insecurely attached patients reported more borderline traits, self-harming behaviors, identity problems and greater emotional dysregulation.

In addition to that, studies have shown a significant association between trauma and a greater comorbidity in adolescents with ED (Jaite et al., 2012). Based on these results, an interesting avenue for future research is to investigate the attachment-related mechanisms by which traumatic experiences, especially childhood maltreatment and interpersonal traumas, might lead to an ED in adolescence. Tasca et al. (2013) examined for the first time in how far attachment insecurity mediates the relationship between childhood trauma and eating pathology in a clinical sample of adults. Their findings indicate that attachment anxiety and avoidance measured with the Experiences in Close Relationships Questionnaire can partly explain this association between trauma and ED. To draw further conclusions, replication of this study using a narrative instrument to assess the unresolved attachment status and also analyze different affect regulation strategies associated with the different attachment patterns is needed (Burns et al., 2012; Danner et al., 2014).

The clinical implications of the preceding issues on attachment in ED affect several practice areas, including the psychological assessment, case formulation and treatment of patients. Assessing a patient’s attachment pattern can not only provide new insights into the symptoms but also helps the clinician to predict the treatment outcome (Buchheim et al., 2012b; Salcuni et al., 2014). To put it differently, assessing different domains of attachment functioning (e.g., interpersonal style, affect regulation, unresolved mental states related to loss or trauma) allows the practitioner not only to address attachment-related goals for treatment, but also provides a template by which differential treatment approaches can be applied for each individual with an ED (for a detailed review, see Tasca, 2014). These new directions might result in better symptom outcomes, a higher treatment completion and an improved long-term interpersonal functioning.

The emerging body of attachment research in patients with ED provides us a promising insight into the interplay between environmental, social and individual factors and how they contribute to the development of this complex and painful condition. Although research in adolescents with ED is still at an early stage, first studies illuminate very interesting details about transgenerational aspects, unresolved trauma and loss, neurophysiological correlates of attachment and its implications for treatment and outcome.

## Summary

The findings of this theoretical review illuminate relevant details about attachment related issues in adolescents with ED and their implications for assessment, case formulation and treatment. In our systematic analysis we found 13 studies using self-report measures of attachment and eight studies employing narrative instruments like the AAI and the AAP in an adolescent and young adult sample. Even though these two approaches measure different facets of attachment, the overall pattern of results from adolescent samples suggest that most of ED patients have insecure attachment. Results regarding links between diagnostic subgroups and the two different insecure attachment styles are not consistent. Some authors found a higher prevalence of the avoidant and others found more anxious individuals among adolescents with ED. The most striking result that emerges from the latest state of narrative-based research is the high prevalence of the unresolved attachment status in adolescent patients and their mothers. Only a small number of studies included fathers and they show that patients feel more alienated from them and they describe them as less caring and more controlling. Furthermore, recent studies demonstrate that adolescents with an unresolved attachment representation have a greater rate of comorbid disorders like PD and depression and higher ED symptom severity. Future studies that investigate traumatizing events, symptom severity and comorbidity in a larger sample of adolescents with ED using a narrative attachment measure might provide a better understanding and treatment of this complex and painful condition.

### Conflict of Interest Statement

The authors declare that the research was conducted in the absence of any commercial or financial relationships that could be construed as a potential conflict of interest.

## Abbreviations

AAI: adult attachment interview
AAP: adult attachment projective picture system
AN: anorexia nervosa
BED: binge eating disorder
BN: bulimia nervosa
ED: eating disorders
EDNOS: eating disorders not otherwise specified
PD: personality disorder
U: unresolved attachment status.
